# Twisted phonon polaritons at deep subwavelength scale

**DOI:** 10.1038/s41377-022-00944-z

**Published:** 2022-08-08

**Authors:** Ren-Min Ma

**Affiliations:** 1 State Key Lab for Mesoscopic Physics and School of Physics, Peking University, Beijing, China; 2 Frontiers Science Center for Nano-optoelectronics & Collaborative Innovation Center of Quantum Matter, Beijing, China

**Keywords:** Optics and photonics, Physics

## Abstract

Hyperbolic polariton vortices carrying reconfigurable topological charges have been realized at deep subwavelength scale.

Photons can carry spin angular momentum (SAM), which is connected to the right-handed and left-handed circular polarizations. In 1992, Allen et al. proposed the orbital angular momentum (OAM) in vortex beams which started a new era of angular momentum studies in optics^1^. Unlike the SAM, OAM occupies an unbounded Hilbert space. Optical vortices carrying non-zero OAM are characterized by a helical phase front and a phase singularity in the beam center. Owing to the carried OAM and phase singularities, optical vortices have been exploited for applications ranging from optical microscopy, communication, micromanipulation, to quantum information processing^2^. Writing in eLight, Andrea Alù and colleagues^3^ have now successfully generated phonon polariton vortices at deep subwavelength scale in the mid-infrared region, which adds a new degree of freedom for on-chip mid-infrared optics.

Polaritons are quasiparticles formed from strong coupling of electromagnetic waves with an electric or magnetic dipole-carrying excitation. Examples of polaritons include phonon polaritons and surface plasmon polaritons, which are formed from coupling of electromagnetic waves with lattice vibration and electron oscillation, respectively. In anisotropic van der Waals (vdW) crystals, phonon polaritons with hyperbolic dispersion can naturally reside because of extreme anisotropy of the atomic interaction between in-plane covalent and out-of-plane vdW bondings^4^. Similar as surface plasmon polaritons, hyperbolic phonon polaritons can carry large wave momenta associated with deeply subwavelength field confinement and, therefore, can be used to strongly enhance light–matter interaction. For surface plasmon polaritons, metallic nanostructures have been employed to generate and manipulate plasmonic vortices on chip^5–8^, which adds a new degree of freedom for various applications. However, it is a long-standing challenge to manipulate hyperbolic phonon polaritons beyond amplitude control.

In their work, Alù and co-authors tackled this challenge by coupling hyperbolic phonon polaritons to a nanostructured metallic spiral disk (Fig. 1). They first fabricated Archimedean spiral shaped gold nanodisks with a thickness of 65 nm by electron beam lithography. They then transferred a hexagonal boron nitride flake with a thickness of ~295 nm on top of the Au nanodisks. A quantum cascade laser with tunable wavelength was employed as the mid-infrared light source to excite hyperbolic phonon polaritons in the hexagonal boron nitride flake through a gold spiral nanodisk, where the edge of the nanodisk supplies additional momentum to convert incident-free-space light into strongly confined hyperbolic phonon polaritons. The nanodisks were shaped into Archimedean spiral for controlling the launched hyperbolic phonon polaritons to be focused to the center of the nanodisks where polariton vortices are formed. The near-field patterns of the generated vortices were recorded by a scattering-type scanning near-field optical microscope.

**Fig. 1:**
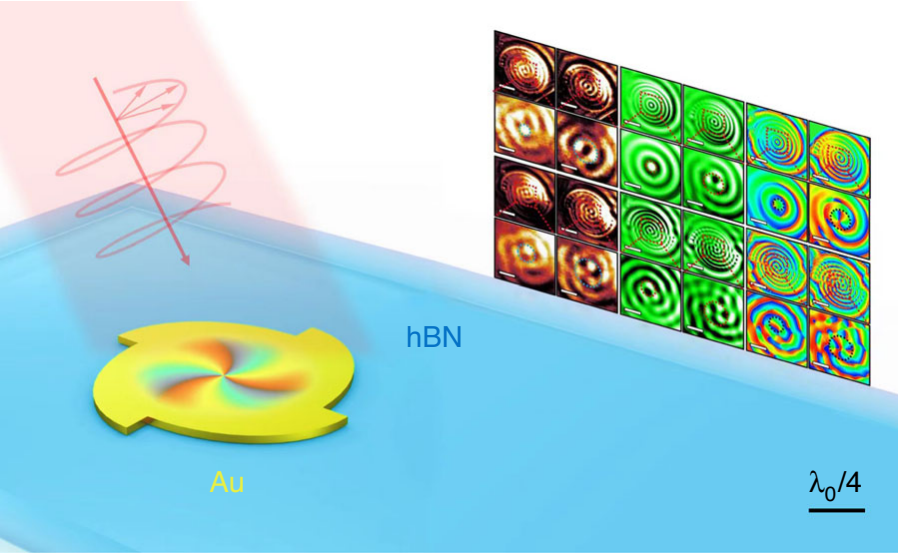
Hyperbolic phonon polariton vortex at deep subwavelength scale. The edge of a gold nanodisk supplies additional momentum to convert incident free-space light into strongly confined hyperbolic phonon polaritons with an in-plane wavelength of λ_0_/8.5 (λ_0_: free space wavelength). The nanodisk is shaped into Archimedean spiral for controlling the launched hyperbolic phonon polaritons to be focused to the center of the nanodisk where a phonon polariton vortex is formed

Remarkably, exotic polaritonic features, including spin–orbit interactions and nanofocusing have been verified in their system at an in-plane wavelength of λ_0_/8.5 (λ_0_: free space wavelength). The authors demonstrated that the topological charge of the excited hyperbolic phonon polariton vortices at deep subwavelengths scale can be simply controlled by the spin of the impinging light due to spin–orbit interactions in the near field. The authors have also demonstrated that polariton vortices are highly tunable using other degrees of freedom, including the geometry of the gold spiral disks, and the hyperbolic features at different wavelength.

The results Alu and colleagues demonstrated are of high importance, because as they added a new degree of freedom for hyperbolic phonon polaritons. The demonstrated hyperbolic phonon polariton vortices in the mid-infrared region, a wavelength region containing important transparency windows of the atmosphere and fingerprint absorption lines of many molecules, have great application potential in super-resolution imaging, ultracompact biological and chemical sensing, and miniaturized polaritonic devices with robust features associated with their topological nature. In future work, it would be interesting to investigate whether dynamically tuning could be realized by other effects, for instance, electro-optic effect. It will also be interesting to see how small the footprint of the hyperbolic polariton vortex generator could be. Apart from passive excitation, active on-chip vortex generators such as vortex quantum emitters or lasers^9–11^ at mid-infrared wavelengths could also be interesting to explore.
